# Clinical research progress in hepatic cirrhosis

**DOI:** 10.1515/jtim-2026-0067

**Published:** 2026-08-24

**Authors:** Yifan Han, Yun Dai, Xiaoyuan Xu

**Affiliations:** Department of Gastroenterology, Peking University First Hospital, Beijing, China

## Introduction

With ongoing advances in research, numerous new findings have emerged in the fields of epidemiology, diagnosis, treatment, and management of cirrhosis. Herein, we provide a commentary on the recent progress in cirrhosis research in 2025.

## Shifts in the etiology of liver cirrhosis

The etiological spectrum of liver cirrhosis has shifted due to the implementation of hepatitis B vaccination, promotion of transfusion safety, and widespread use of antiviral therapy for hepatitis B and direct-acting antivirals for hepatitis C. The proportion of cirrhosis caused by hepatitis B virus (HBV) infection has decreased, whereas the proportions attributable to metabolic-associated fatty liver disease (MAFLD) and alcoholic liver disease (ALD) have increased. Worldwide, the number of cases of MAFLD-related chronic liver disease and cirrhosis rose sharply from 24,841,745 in 1990 to 48,310,981 in 2021. This represents a substantial increase of 94.3% over the years. China currently reports the highest number of cases globally, with a total of 291,247,168 cases, and also records the highest number of new cases annually, amounting to 9,561,143 new cases.^[1]^ Owing to the lack of effective therapies, the burden of MAFLD-induced cirrhosis and hepatocellular carcinoma (HCC) is expected to continue to rise. Clinicians should pay more attention and implement early interventions, such as health education, weight reduction, and pharmacological treatments, to prevent the progression of MAFLD to metabolic dysfunction-associated steatohepatitis (MASH) and cirrhosis.

## Spleen stiffness measurement as a noninvasive assessment of portal hypertension in cirrhosis

Spleen stiffness measurement (SSM) has emerged as an accurate, noninvasive tool for assessing clinically significant portal hypertension (CSPH) in patients with cirrhosis. In a study involving 185 patients with compensated advanced chronic liver disease (cACLD), using an SSM threshold of > 50 kPa, the sensitivity and specificity for diagnosing CSPH were 98.0% and 98.8%, respectively.^[2]^ Moreover, a spleen-dedicated elastography device (100 Hz probe) can improve the diagnostic efficacy of CSPH in patients with cACLD^[2]^ and serves as an excellent tool for predicting CSPH-related complications and risk stratification in this population. The Baveno VII consensus recommends integrating SSM with the Baveno VI criteria (liver stiffness < 20 kPa and platelet count ≥ 150 × 10^9^/L) to identify patients with low-risk varices who can safely avoid endoscopy. The Baveno VI-SSM model, which incorporates SSM, identifies more patients with a low risk of varices than other noninvasive models, with an extremely low risk of decompensation (0.6 per 1000 person-years), thereby avoiding endoscopy in 54.2% of patients and facilitating optimized clinical follow-up and guidance for prophylactic use of non-selective beta-blockers (NSBB).^[3]^ This indicates that SSM can provide adequate risk stratification for selecting treatment candidates. An SSM > 50 kPa can reliably diagnose CSPH in nearly 50% of patients with cACLD, thereby supporting the initiation of NSBB therapy.^[2]^ Furthermore, reductions in SSM during NSBB therapy accurately reflect the treatment response in cirrhotic patients with high-risk varices, with an overall accuracy of 86.4% compared with liver stiffness measurement (LSM).^[4]^ Although SSM performs well for CSPH diagnosis and risk stratification, its role in predicting complications remains limited; integration with machine learning may improve prediction of cirrhosis-related complications.

## Recompensation of liver cirrhosis and its staging

Increasing clinical evidence suggests that liver cirrhosis can achieve recompensation following effective etiological treatment and appropriate management of complications, thereby improving the prognosis of patients. The Chinese Guidelines for Clinical Diagnosis, Treatment, and Management of Cirrhosis recommend classifying recompensated cirrhosis into three stages: temporary (6–12 mo), stable (12–24 mo), and long-term (> 24 mo) recompensation.^[5]^ Temporary recompensation, owing to its brief duration, is associated with an increased risk of recurrent complications and a poor prognosis. Xia *et al*. found that patients with unstable recompensated cirrhosis (*n* = 92) had higher 5-year rates of HCC and all-cause mortality than those with stable recompensation (*n* = 202) (14.7% *vs*. 10.1%, *P* = 0.038), highlighting the need for continued etiological control and timely management of complications.^[6]^ Patients in the stable recompensation phase exhibit a more stable clinical status, higher rates of recompensation, and a reduced risk of complications, although these outcomes are influenced by the underlying etiology and type of the initial decompensating event. Over a 2-year follow-up, recompensation occurred in 62.2% of patients with HBV-related decompensated cirrhosis compared with only 4.15% of those with HCV-related disease, highlighting that timely etiological therapy and effective management of complications are pivotal for achieving stable recompensation.^[7]^ Long-term recompensation is associated with a long-term prognosis similar to that of compensated cirrhosis. Zheng *et al*. reported that in hepatitis B-related HCC patients, 1-, 3-, and 5-year recurrence-free survival (*P* = 0.699) and overall survival (*P* = 0.363) were similar between patients with recompensated and compensated cirrhosis.^[8]^ Patients who achieve long-term recompensation have a prognosis comparable to that of compensated cirrhosis, highlighting recompensation as a key therapeutic goal. Early management of complications, together with sustained etiological and anti-fibrotic therapy, can promote stable and long-term recompensation, improving outcomes in patients with decompensated cirrhosis.

## Long-Term albumin therapy for cirrhosis and relevant complications

International guidelines recommend short-term albumin therapy for acute complications of decompensated cirrhosis, while emerging evidence suggests that long-term albumin therapy (LTA) also provides clinical benefits. In patients with decompensated cirrhosis, the LTA cohort showed significant reductions compared to the non-LTA cohort in the per-patient-per-year incidence of refractory ascites (44.2%), spontaneous bacterial peritonitis (SBP, 52.7%), and hepatorenal syndrome (HRS, 62.6%), suggesting that sustained albumin administration may reduce cirrhosis-related complications. However, whether hepatic encephalopathy can benefit remains controversial compared with the results of the previous ANSWER study.^[9]^

The mechanism by which LTA can improve complications of cirrhosis may be related to the following aspects. First, albumin plays a key role in maintaining oncotic pressure and exerts antioxidant and anti-inflammatory effects. Albumin could have mitigated effective hypovolemia, and this mitigation would explain better control of ascites and related complications, such as renal impairment, HRS type 1, and SBP. Beyond its direct effects, the LTA model, which requires weekly visits, offers significant ancillary benefits through enhanced patient-provider contact, earlier complication detection, improved management, and greater treatment adherence. Considering the negative impact of blood volume expansion caused by LTA treatment on esophageal variceal bleeding and the difficulty in evaluating the efficacy, LTA is currently more suitable for patients with decompensated cirrhosis who also have ascites, SBP, and HRS.

Additionally, compared to standard care, long-term albumin infusion significantly lowers yearly hospital costs for patients with cirrhotic ascites.^[10]^ For patients with decompensated cirrhosis, LTA can not only detect and manage complications in a timely manner but also reduce hospitalization rates, making it a cost-effective treatment option. However, the cost-effectiveness analysis of LTA should be more precise, as it also needs to consider the outpatients, labors, and other indirect costs associated with long-term and frequent follow-ups and should be evaluated according to the healthcare realities and insurance schemes of different countries.

## Conclusion

Overall, synergistic advances in etiological management, noninvasive monitoring of portal hypertension, recompensation, and long-term albumin therapy may reduce cirrhosis-related complications and improve the prognosis.
